# A supertree approach to shorebird phylogeny

**DOI:** 10.1186/1471-2148-4-28

**Published:** 2004-08-24

**Authors:** Gavin H Thomas, Matthew A Wills, Tamás Székely

**Affiliations:** 1 Department of Biology and Biochemistry, University of Bath, 4 South, Claverton Down, Bath BA2 7AY, UK

## Abstract

**Background:**

Order Charadriiformes (shorebirds) is an ideal model group in which to study a wide range of behavioural, ecological and macroevolutionary processes across species. However, comparative studies depend on phylogeny to control for the effects of shared evolutionary history. Although numerous hypotheses have been presented for subsets of the Charadriiformes none to date include all recognised species. Here we use the matrix representation with parsimony method to produce the first fully inclusive supertree of Charadriiformes. We also provide preliminary estimates of ages for all nodes in the tree.

**Results:**

Three main lineages are revealed: i) the plovers and allies; ii) the gulls and allies; and iii) the sandpipers and allies. The relative position of these clades is unresolved in the strict consensus tree but a 50% majority-rule consensus tree indicates that the sandpiper clade is sister group to the gulls and allies whilst the plover group is placed at the base of the tree. The overall topology is highly consistent with recent molecular hypotheses of shorebird phylogeny.

**Conclusion:**

The supertree hypothesis presented herein is (to our knowledge) the only complete phylogenetic hypothesis of all extant shorebirds. Despite concerns over the robustness of supertrees (see Discussion), we believe that it provides a valuable framework for testing numerous evolutionary hypotheses relating to the diversity of behaviour, ecology and life-history of the Charadriiformes.

## Background

The shorebirds and allies (Aves: Charadriiformes; [1]) present an exceptional group for studying numerous evolutionary hypotheses. Their remarkable diversity of social mating system, parental care, sexual dimorphism, ecology and life-history make them an ideal group for unravelling the mechanisms of, for example, sexual selection and sexual conflict. Previous comparative studies have made significant contributions to our understanding of the evolution of mating systems [2], parental care [3,4], sexual size dimorphism [5-7], locomotion and morphology [8], migratory behaviour [9], egg size [10], and plumage colouration [11]. The importance of phylogeny in cross-species comparative studies is well documented [12-14]. Large and well-resolved phylogenies that incorporate divergence times provide powerful tests of a wide range of hypotheses whilst accounting for the effects of shared evolutionary history [13,15]. However, the shorebird studies listed above were limited by the lack of a complete phylogeny for the group. Most of these studies are based on derivations of the seminal work of Sibley and Ahlquist [16], yet this study included less than a quarter of extant and recently extinct shorebird species. Recently extinct taxa (according to Monroe and Sibley [1]) are: the Tahitian sandpiper *Prosobonia leucoptera*, the Canary Islands oystercatcher *Haematopus maedewaldoi*, and the Great auk *Pinguinus impennis*.

**Figure 1:**
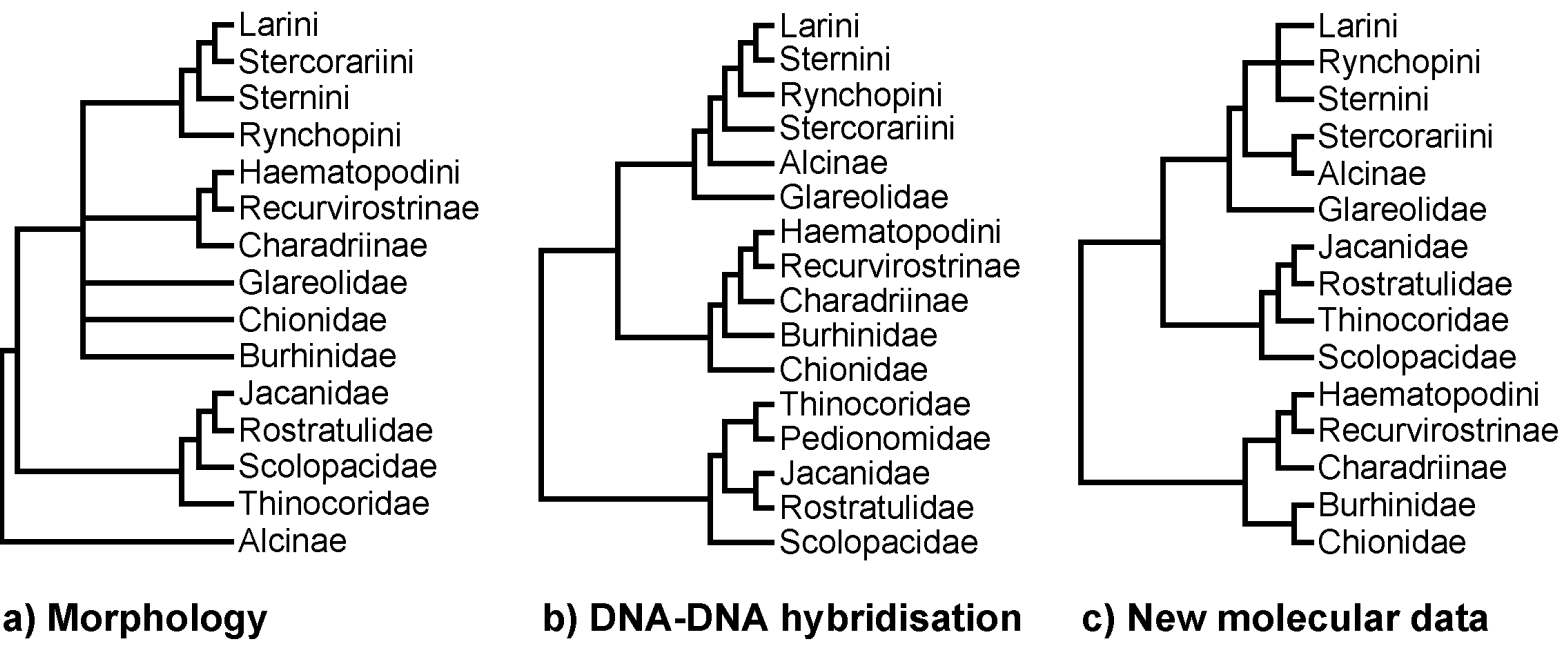
**Previous hypotheses shorebird phylogeny. **Family and subfamily level relationships of shorebirds based on: a) Morphological data [19]; b) DNA-DNA hybridisation [16]; c) Sequence analysis of RAG-1 [20, 21], cytochrome-*b *[22] and myoglobin intron II [21].

Recent molecular studies covering a wide range of shorebird families have drawn attention to conflict in the reconstruction of the deep basal nodes of shorebird phylogeny (figure 1; reviewed by van Tuinen *et al. *[17]). For example, morphological data [18,19] places Alcinae (auks, puffins, murres) at the base of the shorebird tree whilst sequence [20-22] and DNA-DNA hybridisation [16] data suggests that they are a highly derived sister group to Stercorariini (skuas and jaegers), Larini (gulls), Sternini (terns), and Rynchopini (skimmers). It is important to note that taxon coverage differs between these studies and this may be an important factor in determining the tree topology. Specific phylogenies have been derived, for example, for sandpipers [23], the genus *Charadrius *[24], and jacanas [25] using DNA sequence data. In contrast, morphological evidence provided the basis for Chu's [26] study of gull phylogeny. Strauch [18] presented the most complete data set of 227 Charadriiformes species. However, despite the plethora of cladograms for particular shorebird groups (see reviews by Sibley and Ahlquist [16]; Thomas *et al. *[22]), those that address relationships across the whole clade use either sparse taxon sampling [16,27], or are based on reassessments of Strauch's [18] data [19,28-30]. Note that Dove [30] included a feather microstructural analysis in addition to her reanalysis of Strauch's [18] data.

### Combining phylogenetic data

Numerous methods and types of data can be used to infer phylogeny. Frequently, as in Charadriiformes, a single analysis incorporating all taxa of interest is absent. Under the principle of total evidence [31], all sources of phylogenetic information should be combined to maximize their explanatory power. Eernisse and Kluge [32] define total evidence as a method for seeking the best fitting phylogenetic hypothesis for an unpartitioned set of synapomorphies (shared derived characters) using character congruence (characters combined in a supermatrix). Hence, this method combines the primary data (molecular, morphological and behavioural characters) into a single analysis. The approach is powerful because weak signals in the partitioned data sets may be enhanced when combined, and previously obscured relationships may be revealed [33].

The total evidence approach has both practical and theoretical problems. First, only certain types of data can be combined. For example, nucleotide sequences and morphological traits can be readily assessed together as characters, but it is not generally possible to include nucleotide sequences and genetic distance data in a single analysis [34]. We acknowledge that Lapointe *et al. *[35] suggest a distance based approach to combine otherwise incompatible data in a total evidence analysis, although this method has not been tested beyond a single application. The consequence is that it is rarely possible to combine all sources of data in practice and the lack of overlap in combinable data sets may result in a reduction of the number of taxa included. Second, Miyamoto and Fitch [36] contend that combining data sets is rarely justified because partitions of phylogenetic data are real and unequivocal. They argue that several partitions producing similar topologies provide multiple lines of independent evidence supporting that topology.

Theoretical arguments over the benefits of total evidence will undoubtedly continue, but perhaps the major barriers to its use are the often very high computational demands of large matrices, and the *a priori *exclusion of certain data types. This is particularly true of Charadriiformes phylogeny, where one of the most significant contributions to the field – DNA-DNA hybridisation – cannot be included. An alternative set of techniques, collectively termed supertrees (e.g., Matrix Representation with Parsimony, MRP; [37,38]), enables combination of trees (rather than raw data) from otherwise incompatible sources. MRP methods code source phylogenies based on the presence and absence of taxa at each node of the tree [37-39] and are thus one step removed from the primary data. It is important to recognise that supertrees should not be regarded as a replacement for exhaustive phylogenetic studies of the primary data and there are drawbacks to the methods (see Discussion). However, they do enable very large phylogenies to be constructed rapidly [15]. Supertrees have been constructed successfully for a wide variety of taxa including carnivores [15], primates [39], seabirds [40], dinosaurs [41], and grasses [42].

Shorebirds are particularly well suited for supertree treatment, since there are numerous incomplete phylogenies available and a broader phylogeny is desirable to facilitate powerful analyses of numerous evolutionary hypotheses (see above). Here, we present the first complete composite phylogeny of extant and recently extinct [1] shorebirds using the MRP approach. We are therefore combining data on tree topologies, and not conducting a simultaneous analysis on the original data. We also use fossil and molecular data to estimate divergence times (see Methods). The combination of complete taxonomic coverage and the inclusion of branch lengths provide the basis for future comparative analyses of Charadriiformes evolution. In addition, conflicting and unresolved areas of Charadriiformes phylogeny are revealed.

## Results and Discussion

### Supertree resolution and topology

**Figure 2:**
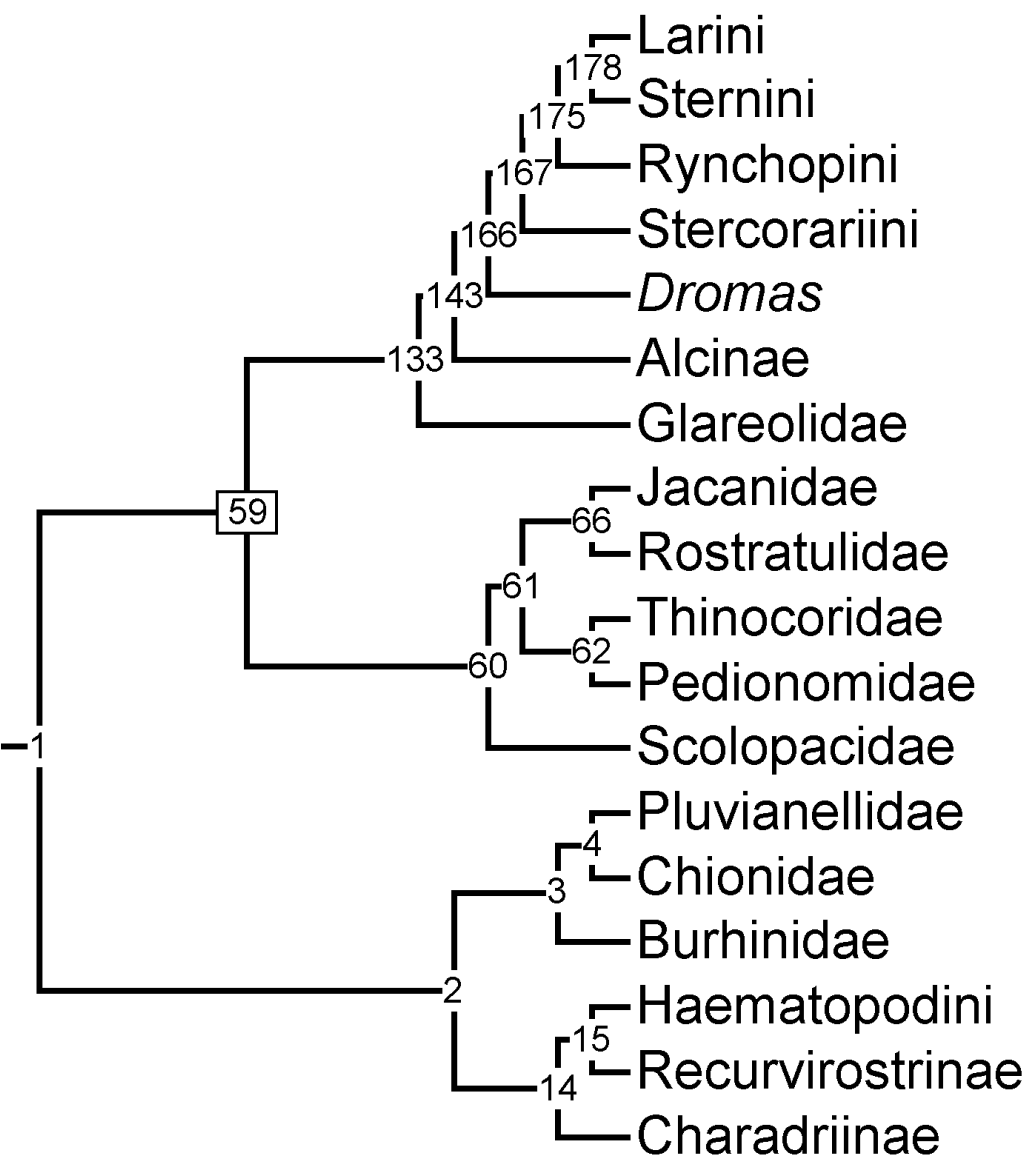
**Summary of shorebird supertree. **Family and subfamily level relationships of shorebirds based on 50% majority rule tree. Numbers on nodes refer to age estimates in additional file 1. Boxed node numbers indicate that node collapses to its immediate ancestor in the strict consensus tree (see also additional files 2 and 3 for the full 50% majority rule and strict consensus trees respectively).

**Figure 3:**
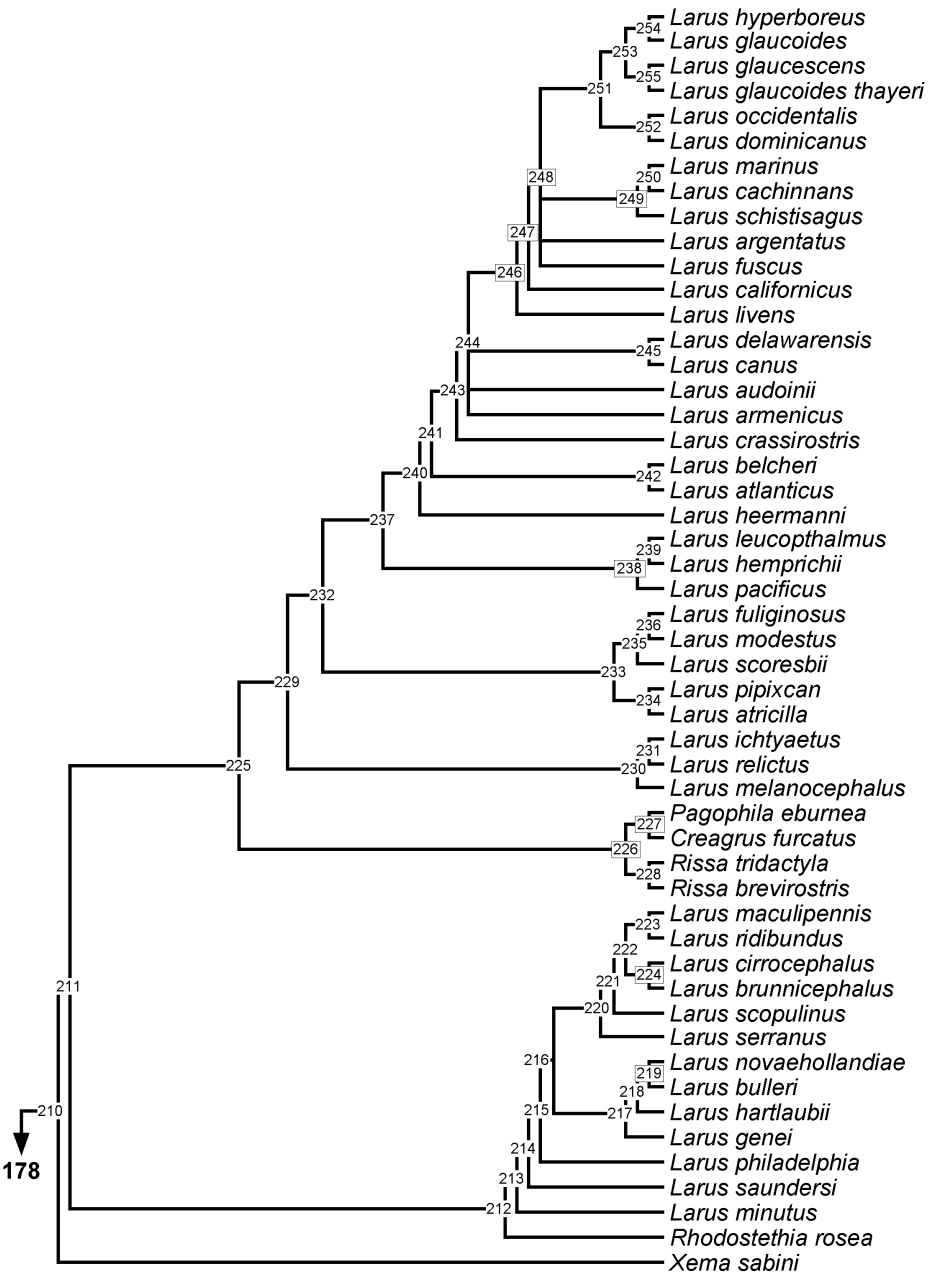
**Phylogeny of Larini. **50% majority rule supertree showing the relationships of the Larini. Numbers on nodes refer to age estimates in additional file 1. Boxed node numbers indicate that node collapses to its immediate ancestor in the strict consensus tree (see also additional files 2 and 3 for the full 50% majority rule and strict consensus trees respectively).

**Figure 4:**
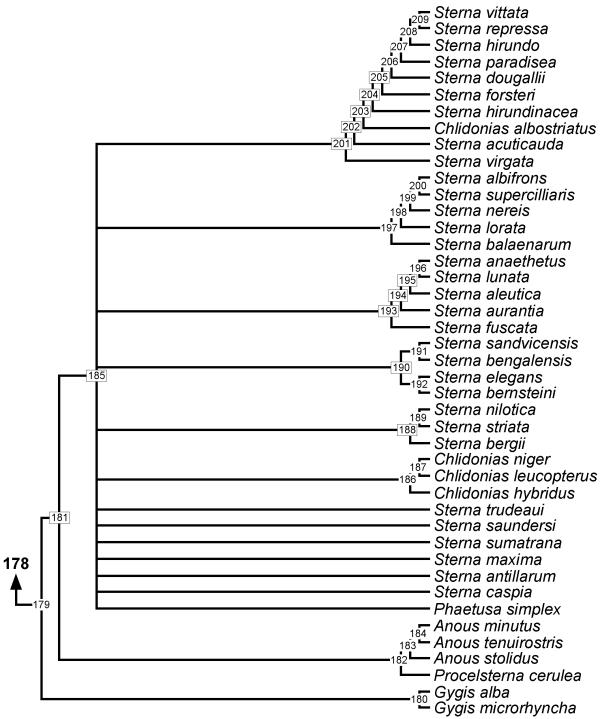
**Phylogeny of Sternini. **50% majority rule supertree showing the relationships of the Sternini. Numbers on nodes refer to age estimates in additional file 1. Boxed node numbers indicate that node collapses to its immediate ancestor in the strict consensus tree (see also additional files 2 and 3 for the full 50% majority rule and strict consensus trees respectively).

**Figure 5:**
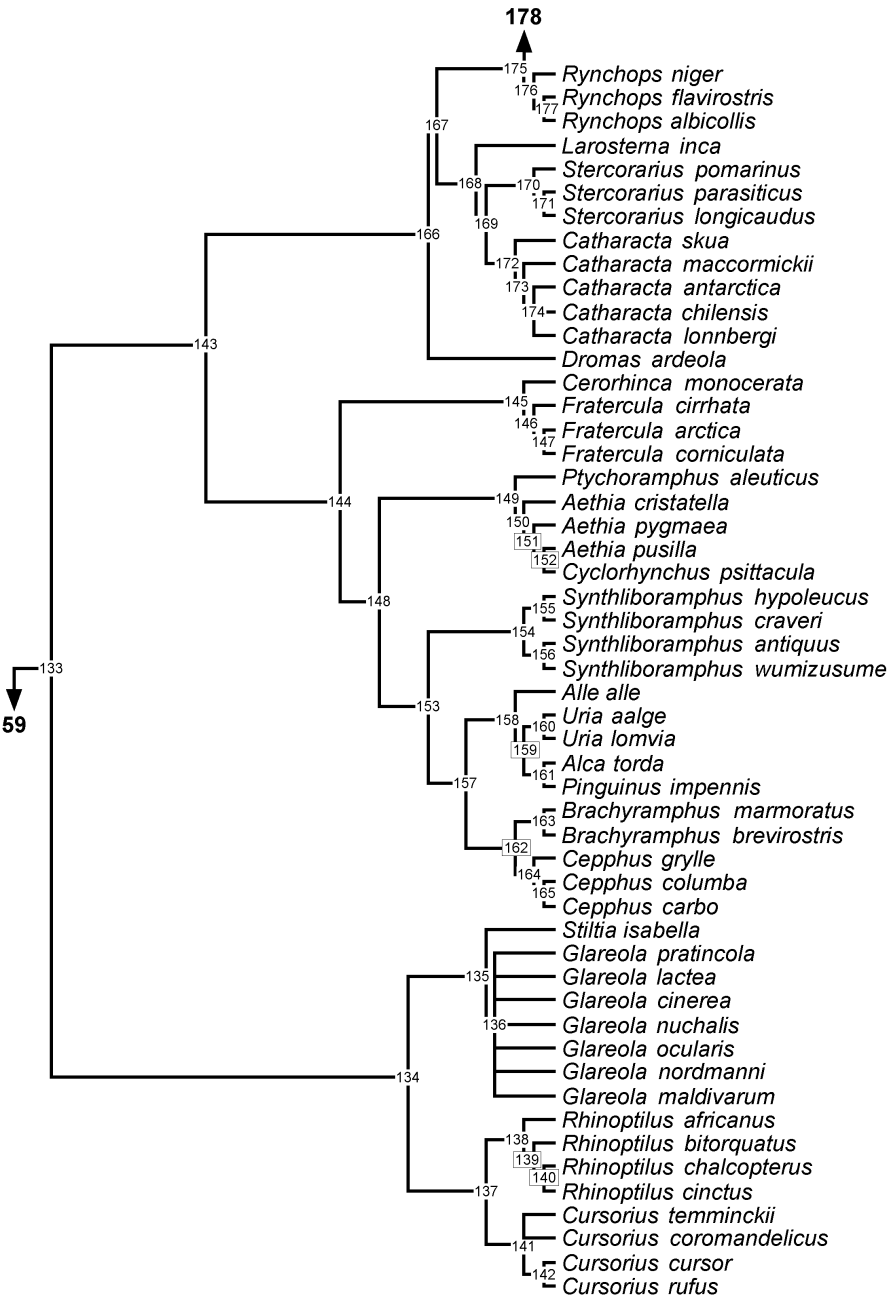
**Phylogeny of Rynchopini, Stercorariini, *Dromas*, Alcinae, and Glareolidae **50% majority rule supertree showing the relationships of the Rynchopini, Stercorariini, *Dromas*, Alcinae, and Glareolidae. Numbers on nodes refer to age estimates in additional file 1. Boxed node numbers indicate that node collapses to its immediate ancestor in the strict consensus tree (see also additional files 2 and 3 for the full 50% majority rule and strict consensus trees respectively). Node numbers 139 and 140 have no support from any source tree and are novel clades.

**Figure 6:**
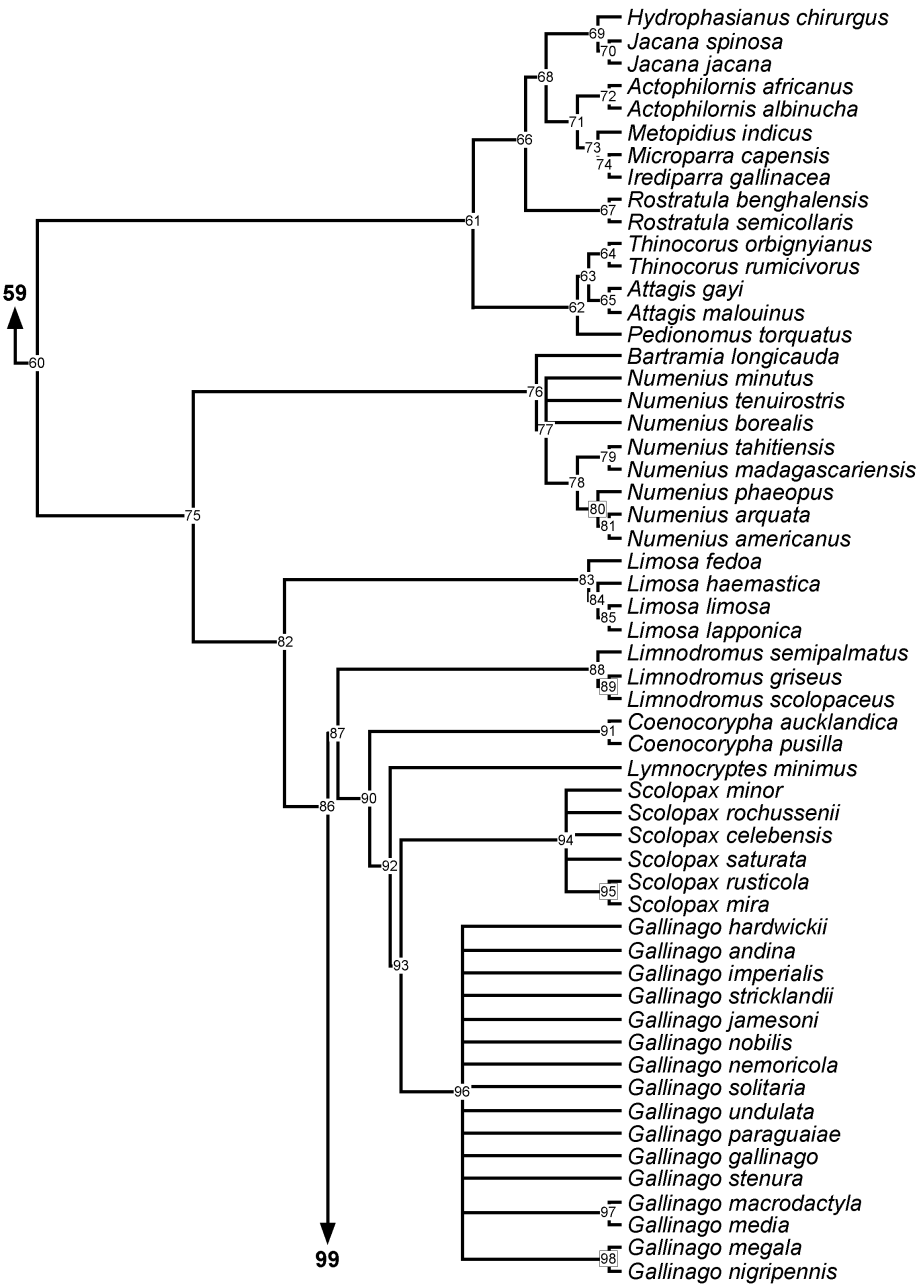
**Phylogeny of Jacanidae, Rostratulidae, Thinocoridae, Pedionomidae and Scolopacidae **50% majority rule supertree showing the relationships of the Jacanidae, Rostratulidae, Thinocoridae, Pedionomidae and Scolopacidae. Numbers on nodes refer to age estimates in additional file 1. Boxed node numbers indicate that node collapses to its immediate ancestor in the strict consensus tree (see also additional files 2 and 3 for the full 50% majority rule and strict consensus trees respectively). Node numbers 85 and 89 have no support from any source tree and are novel clades.

**Figure 7:**
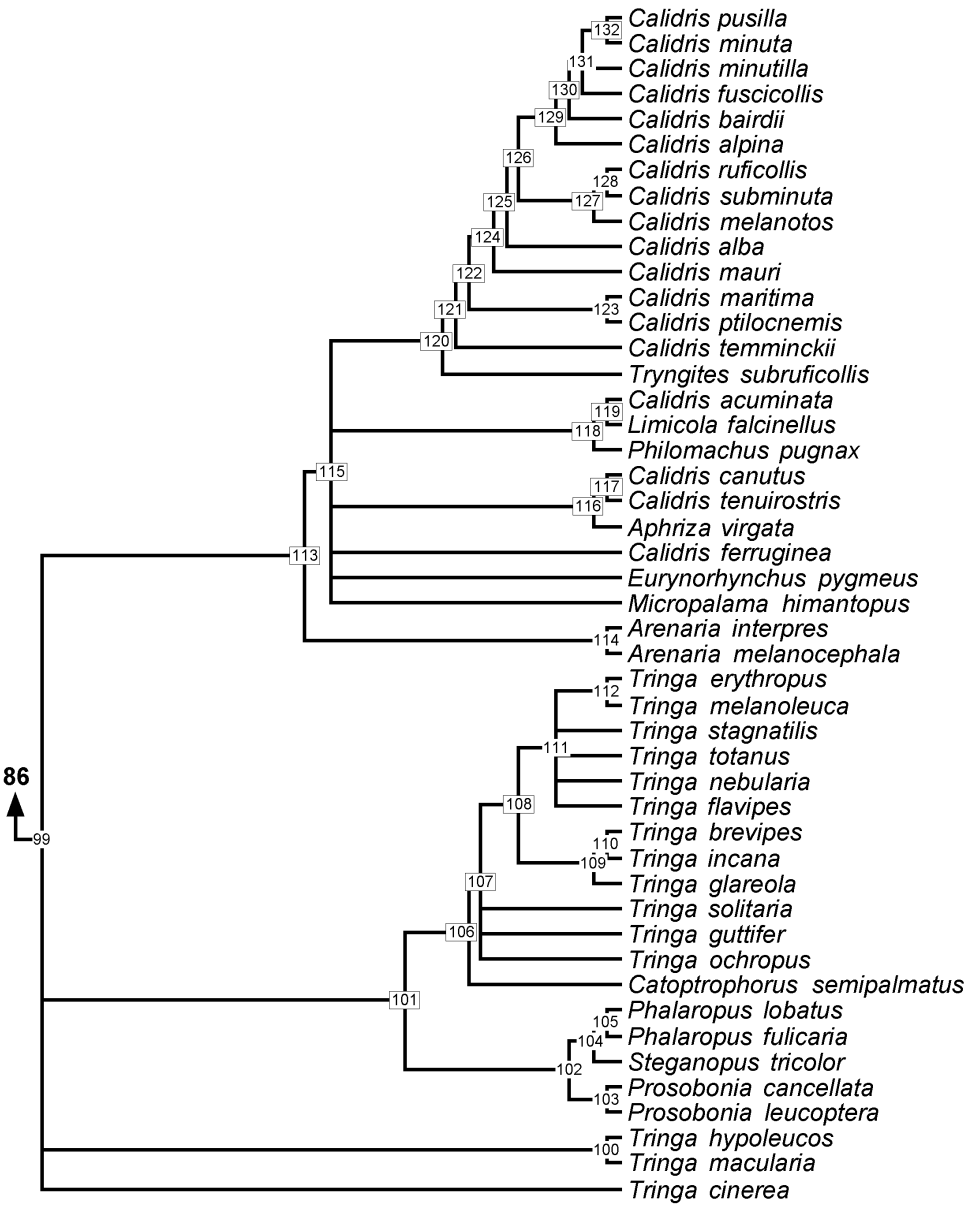
**Phylogeny of Scolopacidae **50% majority rule supertree showing the relationships of the Scolopacidae. Numbers on nodes refer to age estimates in additional file 1. Boxed node numbers indicate that node collapses to its immediate ancestor in the strict consensus tree (see also additional files 2 and 3 for the full 50% majority rule and strict consensus trees respectively). Node numbers 108 and 122 have no support from any source tree and are novel clades.

**Figure 8:**
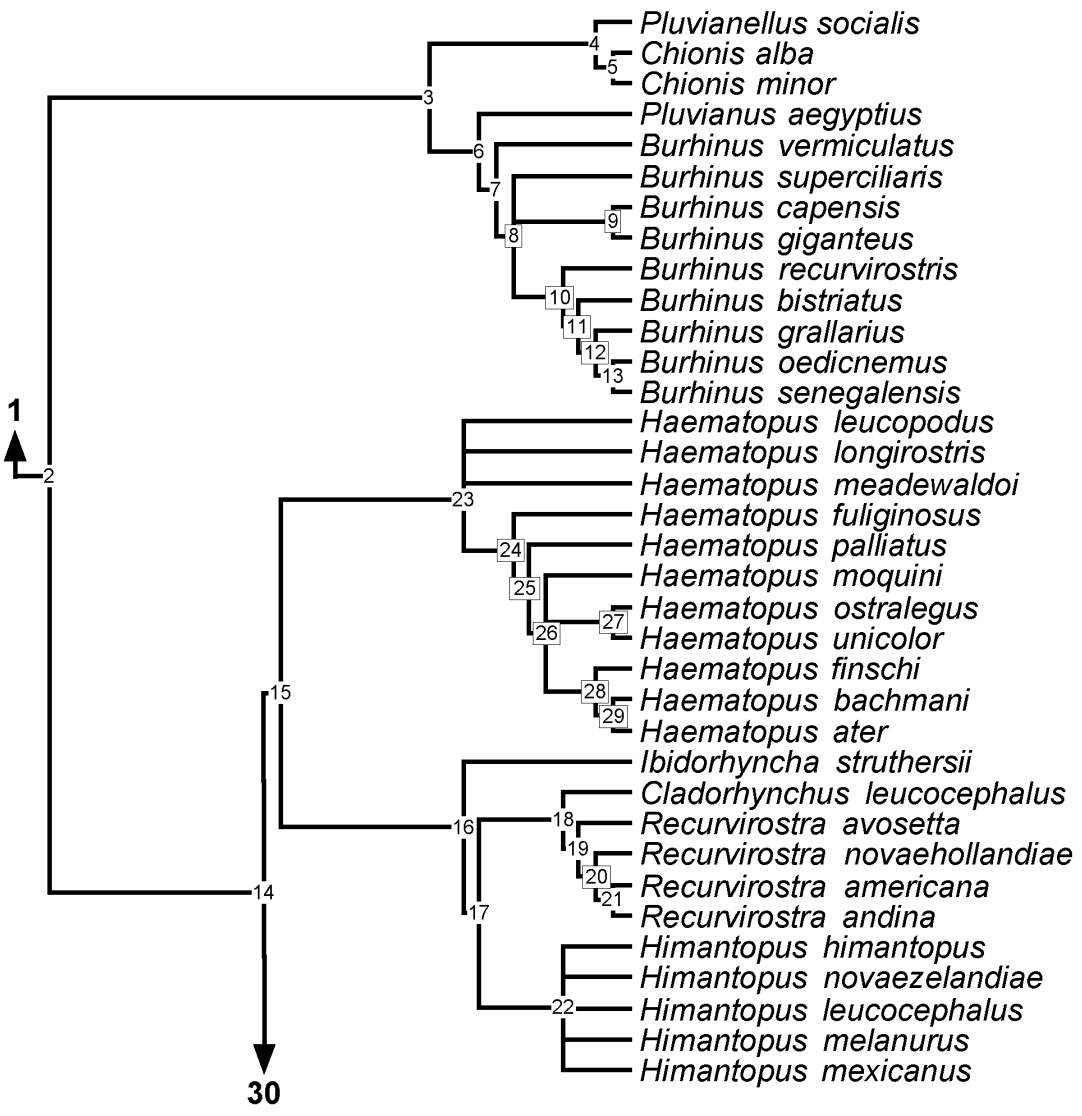
**Phylogeny of Pluvianellidae, Chionidae, Burhinidae, Haematopodini and Recurvirostrini **50% majority rule supertree showing the relationships of the Pluvianellidae, Chionidae, Burhinidae, Haematopodini and Recurvirostrini. Numbers on nodes refer to age estimates in additional file 1. Boxed node numbers indicate that node collapses to its immediate ancestor in the strict consensus tree (see also additional files 2 and 3 for the full 50% majority rule and strict consensus trees respectively). Node numbers 20 and 29 have no support from any source tree and are novel clades.

**Figure 9:**
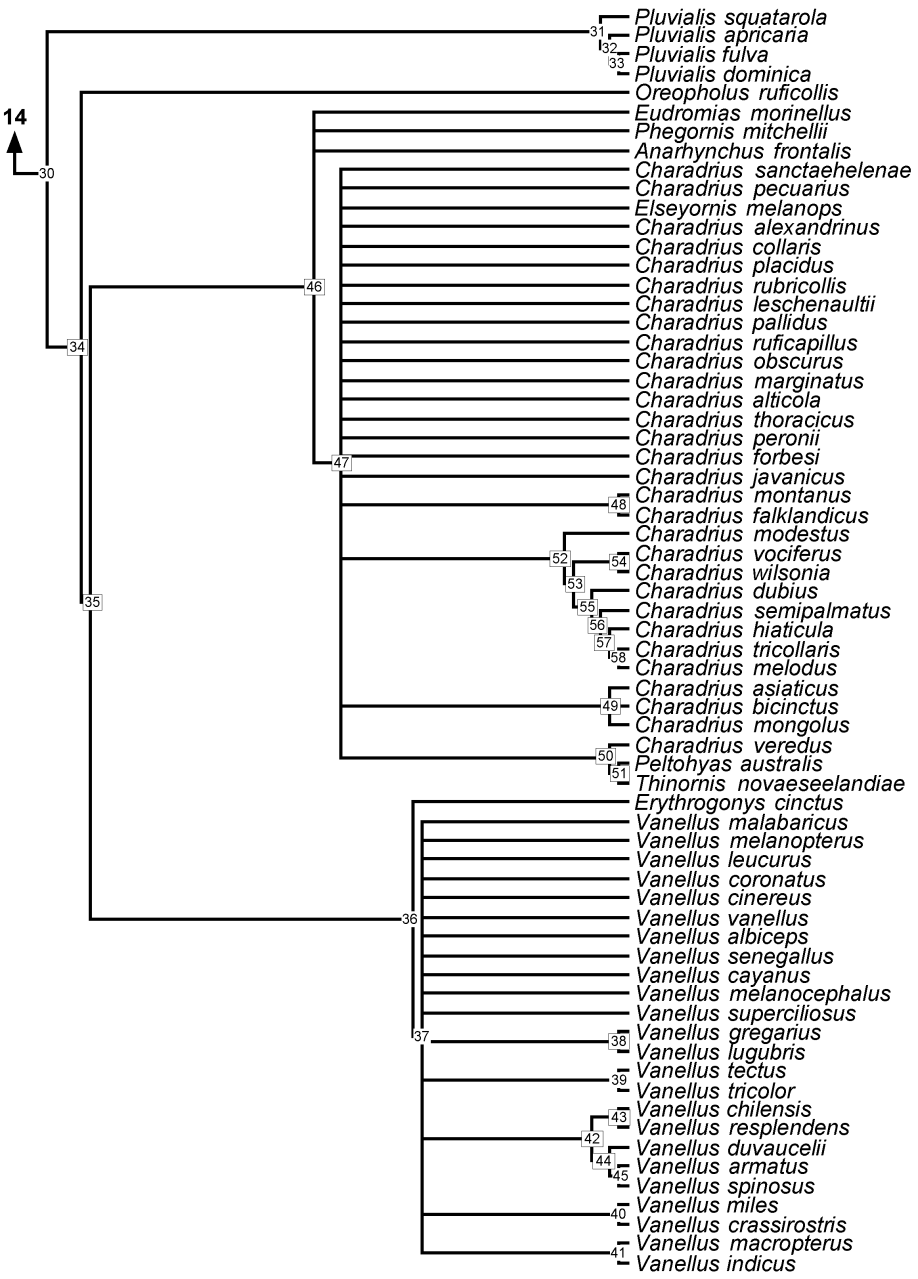
**Phylogeny Charadriinae **50% majority rule supertree showing the relationships of the Charadriinae. Numbers on nodes refer to age estimates in additional file 1. Boxed node numbers indicate that node collapses to its immediate ancestor in the strict consensus tree (see also additional files 2 and 3 for the full 50% majority rule and strict consensus trees respectively). Node number 57 have no support from any source tree and are novel clades.

We found 1469 equally short trees of length 1847 steps using the parsimony ratchet approach (see Methods). This compares favourably to a standard heuristic search that yielded shortest trees of 1853 steps. All subsequent results and discussion refer to the parsimony ratchet analyses. Figure 2 shows the family and subfamily level relationships of shorebirds based on the strict and 50 % majority-rule consensus tree (see additional file 1 for branch length estimates). Figures 3,4,5,6,7,8,9 show the species level phylogeny. The full 50% majority rule consensus and the strict consensus trees are available as additional file 2 and 3 respectively. The 50% majority-rule consensus tree is well resolved (73.1%; 255 nodes out of a possible 349 in a fully bifurcating tree), although the strict consensus tree is only 49.6% resolved (173 from 349 possible nodes). The majority rule tree includes nine novel clades (numbers 20, 29, 57, 85, 89, 108, 122, 139, 140) that do not appear in any of the source trees; all of these occur towards the tips of the tree. This is a general problem in supertree construction and such clades should be collapsed as they have no support [41]. To demonstrate where the MRP method has performed badly we have included the novel clades in all figures and list details in the figure legends. In addition, 58 nodes are supported by only one character (see additional file 1). Each of these nodes is left over from a single source tree. Assessing the support for such nodes is problematic because this may simply reflect a lack of research directed at the taxa in question. A major challenge for supertree construction is to develop measures of support that reflect the robustness of nodes in the source trees. We list the number of characters supporting each node (additional file 1) but stress that these are not measures of tree robustness and may not be directly comparable even within the same tree. This is because the taxon coverage across source trees is highly variable so some nodes have more potential support than others. Furthermore, because measures of support used in the source trees differ between studies (some source trees include no measures of support), it is impractical and of dubious value to use these measures to assess the robustness of the supertree.

The majority of unresolved nodes in the shorebird supertree are located towards the tips of the phylogeny. For example, the genus *Gallinago *forms a monophyletic clade but only two pairs of species are resolved from 14 species (*G. megala *and *G. negripennis*; *G. macrodactyla *and *G. media*) in the majority-rule tree. Only the latter relationship remains in the strict consensus tree. In addition, clades including the genera *Charadrius *and *Vanellus*, *Calidris *and *Tringa*, *Sterna*, and *Scolopax *are poorly resolved. This may reflect a bias in phylogenetic studies of shorebirds. For instance, we found six source trees for Alcinae [43-48] but none devoted to *Scolopax *or *Gallinago*. Thomas *et al. *[49] indicate that this may be a problem for shorebird studies in general and reported a strong skew favouring research on northern hemisphere species.

In contrast to the within genera relationships, the generic and family levels are generally well resolved. The supertree indicates three monophyletic Charadriiformes lineages (figure 2). Family and subfamily resolution within each lineage is high, however the relative position of each group is unresolved in the strict consensus tree. This is an important point because the deepest relationships of shorebird phylogeny are contentious [22]. The 50% majority-rule consensus tree indicates that the gulls and allies (Larini, Sternini, Rynchopini, Stercorariini, *Dromas*, Alcinae, and Glareolidae) are sister to the sandpipers and allies (Scolopacidae, Jacanidae, Rostratulidae, Thinocoridae, Pedionomidae). The most basal lineage includes the plovers and allies (Charadriinae, Pluvianellidae, Chionidae, Burhinidae, Haematopodini and Recurvirostrini). The gulls and allies clade is most consistent with DNA-DNA hybridisation [16], indicating that Larini are sister to Sternini and that Rynchopini are sister to this group. This conflicts with morphology-based topologies where Stercorariini are sister to Larini and Sternini with Rynchopini basal to both. Indeed, the position of Stercorariini remains controversial and most recently they were placed as sister to Alcinae [20-22]. In contrast, morphological evidence [18,19] places Alcinae at the base of the whole Charadriiformes tree with Stercorariini sister to Larini. Thus, the position of Alcinae is uncertain and appears to be dependent on the type of data, with fundamental differences between molecular based analyses and morphological analyses. The taxon sampling of previous morphological and molecular studies varies considerably and it may be this, rather than genuine differences in the phylogenetic signal of different data types, that is the cause of conflict in resolving the phylogenetic position of Alcinae. However, it is encouraging that van Tuinen *et al. *[17] suggested that new unpublished osteological data are consistent with the more derived position indicated by molecular data. The supertree resolves Glareolidae outside the Larini, Sternini, Rynchopini, Stercorariini, *Dromas*, Alcinae clade. This is also the case with recent molecular and previous DNA-DNA hybridisation studies. Morphological studies have failed to resolve the position of Glareolidae, placing the family in a large polytomy with all other major groups except Alcinae and the sandpipers and allies (fig. 1). A novel development in shorebird phylogeny is the placement of the black-rumped buttonquail *Turnix hottentotta *as a sister to the gulls and allies (Larini, Sternini, Rynchopini, Stercorariini, *Dromas*, Alcinae, and Glareolidae) based on the nuclear RAG-1 gene [20]. We did not include this species in the supertree because to date Paton et al. [20] remains the only study to reveal an apparently robust relationship. More diverse sampling of the buttonquails (Turnicidae) is essential to corroborate the general affinities of this family.

The relationships within the plover clade appear to be reasonably stable. Morphological, molecular, and DNA-DNA hybridisation all place Charadriinae as sister to Haematopodini and Recurvirostrini; our supertree is consistent with these relationships. However, it is not clear whether Burhinidae and Chionidae are sister to each other [20-22] or whether Chionidae are sister to a Charadriinae, Haematopodini, Recurvirostrini, and Burhinidae clade [16]. Our supertree also included Pluvianellidae, a family consisting of only one species (magellanic plover *Pluvianellus socialis*) and places this as sister to Chionidae. If Pluvianellidae are excluded, the supertree is consistent with the sister group relationship of Burhinidae and Chionidae.

The sister group relationship of Jacanidae to Rostratulidae is well established [16,18-22] and is found in our supertree. The supertree resolves the Thinocoridae and Pedionomidae as sister taxa and this group is sister to the Jacanidae and Rostratulidae. The large Scolopacidae clade is at the base of the sandpiper clade consistent with recent molecular studies [20-22] and the DNA-DNA hybridisation tapestry [16].

Taken together, it is evident that the supertree is generally more consistent with molecular data (both recent sequence studies and DNA-DNA hybridisation) than with analyses based on morphology. However, it is of course possible that this reflects the greater number of molecular source trees available rather than indicating that molecular data is actually better at resolving shorebird phylogeny. We included several large morphological phylogenies [e.g [18,19,26,30,43]] but the majority of source trees (29 out of 51) were based on molecular evidence (see additional file 5).

### Node dates

The higher resolution of the majority-rule tree means it is more likely to be of use in comparative studies. We therefore estimated node ages for this topology only (see additional file 1 and 2). We stress that our estimates of node dates are a first attempt at dating the whole tree and have several limitations. First, the fossils used to calibrate seven nodes in the tree are unlikely to be the earliest members of their respective families thus these dates will be underestimates. Second, we assumed that the fossils are grouped with the extant members of the family but this requires formal testing in a phylogenetic framework. Third, the pure birth model assumes that no extinction occurs but this may be unrealistic and it is likely that extinction processes have reduced the representation of older lineages [15]. Furthermore, this model is derived from the topological structure of the tree so errors in tree reconstruction will likely lead to errors in branch length estimation. However, this approach has been employed previously in supertrees of primates [39] and carnivores [15] explicitly to facilitate comparative analyses. Despite these caveats, simulation studies have demonstrated that comparative methods such as independent contrasts are robust to errors in branch length [50] and no viable alternative for dating supertrees has been proposed. Nonetheless, we urge that alternative branch length assumptions are explored if the shorebird supertree is used in future comparative studies. At present, the calibrated RAG-1 tree of Paton et al. [20] remains arguably the most thorough and reliable measure of divergence times for Charadriiformes.

A fuller understanding of the phylogenetic affinities of fossil shorebirds will probably improve estimates of node ages for the group. For example, the extinct form Graculavidae, is represented by fossils from the Maastrichtian of New Jersey [51] and Cretaceous of Wyoming [52] but its position within the shorebird clade is unclear. Feduccia [53] suggests that it may be basal and a formal corroboration of this would support proposals for a late Cretaceous origin of shorebirds. The difficulties in dating the shorebird tree are further illustrated by fossil representatives of Recurvirostrini and Burhinidae which are much older than current estimates suggests. The earliest record of the Recurvirostrini is estimated to be over 50 million years old [54] whilst recent discoveries of a possible member of the Burhinidae are dated to around 70 mya [55,56]. There is clearly a need for an integrated phylogenetic study including both extinct and extant shorebirds.

### Supertree bias

Supertrees are still at an early stage of development and many aspects of MRP, and supertree methods in general, are not yet clearly understood. Steps can be taken to ensure that the supertree includes the most appropriate sets of sources trees, such as only using trees from explicitly phylogenetic studies. This is not always straightforward and could result in the exclusion of important information. For instance, in our shorebird supertree, we included Sibley and Ahlquist's DNA-DNA hybridisation tapestry [16] although this is based on distance measures rather than more rigorous phylogenetic methods. Even if very strict tree selection criteria are applied, there are still likely to be biases in the data set. For example, not all source trees are equally well supported, yet in most supertree analyses each tree is treated equally [57]. This is a problem for supertree construction because whilst it is theoretically possible, and indeed beneficial, to weight source trees based on support values [57] it is rarely possible in practice. Many source trees do not have support values and those that do may use different methods, (e.g, bootstrapping or decay indices) which cannot be directly compared with each other. An additional problem that has not been fully resolved relates to correlations between source trees [58]. Several source trees based on the same data set may unduly increase the influence of that data set on the supertree analysis. However, there is no formal way of determining how much overlap to allow and the choice of source trees that go into supertree construction inevitably involves some degree of subjective reasoning. For the shorebird supertree we used strict Reduced Cladistic Consensus trees to summarise potential source trees that were from the same data set but based on different methods. For example, Thomas *et al. *[22] based their phylogeny on cytochrome-*b *but used a range of methods including parsimony and Bayesian analyses. We therefore combined these trees to minimise bias. In contrast, Ericson *et al. *[21] used two types of data: sequences from the nuclear RAG 1 gene and sequences from the myoglobin intron II. They carried out three analyses: each gene separately and then the two combined in a single analysis. In this case, we used three source trees. It could be argued that the combined analysis of Ericson *et al. *[21] should be excluded because of the possible overlap with the individual analyses. However, under the principle of total evidence, the combined data set may result in novel relationships being revealed [31,33] and therefore could contribute important information to the supertree. Simulation and empirical studies are required to fully understand these and other possible biases in supertree construction (e.g., the influence of source tree size and shape) and formal protocols for the selection of source trees are desirable. For transparency, we include a summary of the source trees used, data type, and the main taxa included in the study (additional file 5).

Our shorebird supertree is highly consistent with recent advances in the molecular phylogenetics Charadriiformes. However, we urge caution when using the tree in comparative analyses and encourage the additional use of alternative phylogenies and branch length assumptions. It is particularly important to note that the position of some groups such as the Alcinae remains controversial and that although the majority rule tree is consistent with recent molecular studies, the strict consensus tree fails to resolve the deepest nodes.

## Conclusions

The supertree presented here is, to our knowledge, the first attempt to reconstruct the phylogeny of the entire order Charadriiformes. Overall, the supertree is highly consistent with recent molecular hypotheses of shorebird phylogeny. However, it is apparent that fresh attempts to resolve both the phylogeny and estimates of age will be dependent on further gene sequencing and new fossil discoveries. The affinities of the Alcinae and the relationships between the three major shorebird clades require further corroboration, and studies of several genera such as *Gallinago *and *Vanellus *are desirable. Furthermore, additional work is required to establish the true affinities of the Turnicidae. Nonetheless, it appears that shorebird phylogeny is gradually approaching a consensus view. The broad taxonomic scope and consistency of the supertree mean that is of potentially great value to future comparative studies (accepting the caveats discussed above) of the behaviour, life-history, ecology and conservation of this diverse group.

## Methods

### Supertree construction

Possible source trees were identified from online searches of Web of Science  covering the years 1981 to 2004. We used the single key strings phylogen*, cladistic*, clado*, classif*, systematic*, and taxonom* (where the asterisks allow variations such as "phylogeny" or "phylogenetics") in the topic field, in conjunction with a major Charadriiformes taxon name (scientific or common). As supertree methods have been criticized for being biased towards historical trends, we preferred those studies that explicitly set out to derive a phylogenetic hypothesis and so exclude purely (and typically older) descriptive taxonomic works. The Sibley and Ahlquist [16] DNA-DNA hybridisation tapestry may be viewed as non-cladistic, but it was clearly the authors' intention to reconstruct the phylogeny of birds. Furthermore, it provided a vital catalyst for subsequent studies of avian (including shorebird) phylogeny. We therefore included the DNA-DNA hybridisation hypothesis as a source tree in our analyses. Simulation studies have demonstrated that the performance of supertree methods is improved by including at least one taxonomically complete (or near complete) source tree [57]. We therefore make an exception to our self-imposed rule, and in addition use the taxonomic hierarchy of Monroe and Sibley [1] as a source tree as this includes all extant Charadriiformes species. We acknowledge that this taxonomy is based largely on Sibley and Ahlquist's [16] DNA-DNA hybridisation tapestry.

The initial search identified 78 source trees from 44 publications. Each source tree was typed as a text file in Nexus format [59]. We coded trees to the species level with species names taken from Monroe and Sibley [1], but note that *contra *Monroe and Sibley [1], we use Charadriiformes not Charadrii to refer to the whole group. Several studies included the gull *Larus thayeri *[26,60-63] either as a subspecies of *Larus glaucoides *(*Larus glaucoides thayeri *in Monroe and Sibley [1]) or a species in its own right. In recognition of this, we included *Larus glaucoides thayeri *as the only subspecies in our data set thus increasing the total taxa to 366. Monroe and Sibley [1] include 16 species of the family Pteroclidae within the Charadriiformes. However, the relationship of this family to the Charadriiformes is uncertain and they have recently been placed in their own order [64]. We include the Pteroclidae in our analyses only as a means of rooting the tree. Where there were multiple most parsimonious trees (MPTs), or where source trees had been derived from predominantly overlapping data (e.g., from the same data but using alternative methods), we used RadCon [65] to produce strict Reduced Cladistic Consensus trees (RCC [66,67]). The output is in the form of a reduced consensus profile and from this we selected the tree with the highest Cladistic Information Content (CIC) [65,68]. This resulted in a total of 51 source trees from which our supertree is derived and these are summarised in additional file 5.

We produced an MRP matrix of the 51 Nexus [59] source trees in RadCon [65] (see additional file 6 for the MRP file). We used the original MRP coding method of Baum [37] and Ragan [38]. Weighting source trees based on node support such as bootstrapping improves the accuracy of MRP supertrees [57]. However, this is only possible if all source trees can be weighted on the same criteria [57]. The absence of branch support measures in many of the shorebird source trees precludes this approach from the present study; hence, subsequent analyses were conducted using equally weighted parsimony.

The tendency of large data sets to produce many sub-optimal trees that are close in length and topology to the shortest tree is a serious problem in phylogenetics. Standard heuristic searches frequently are trapped searching within globally sub-optimal "islands" and the tree search is often aborted before completion. Nixon [69] proposed a new method to avoid this problem. The "Parsimony Ratchet" reweights a random set of characters from the data set. This may result in the tree island no longer representing a local optimum and the heuristic search continues until a new optimum is reached. The algorithm then reverts to the original weighting and the search continues. Nixon [69] demonstrated the efficacy of the method on a 500-taxon data set, where the ratchet-based search found a tree two steps shorter than standard heuristic searches. We used PAUPRat [70] to implement a parsimony ratchet in PAUP* [59]. The default settings of 200 iterations and 15% perturbation of characters for reweighting were used and we carried out 20 replicates. Equally parsimonious trees were summarized using both strict and 50% majority-rule consensus methods.

We did not calculate any measures of branch support for two reasons. First, their validity and meaning is questionable in MRP supertrees [41]. Second, the number of taxa included in our data set is too large to allow practical calculation of any branch support indices (e.g., decay indices [71]) on a desktop computer.

### Dating the supertree

Following Purvis [39] and Bininda-Emonds *et al. *[15] we dated the supertree using both absolute and relative dates. We used data from the Fossil Record 2 [54] as the source of fossil-based absolute dates. This yielded estimates for Jacanidae (*Nupharanassa tolutaria*, Rupellian), *Phalaropus *(*Phalaropus elenorae*, Middle Pliocene), Burhinidae (*Burhinus lucorum*, Lower Miocene), Glareolidae (*Paractiornis perpusillus*, Lower Miocene), Alcinae (*Petralca austrica*, Rupellian), Stercoariini (*Stercorarius *sp., Middle Miocene), and Larini (undetermined, Rupellian). We took the midpoint of the range from the Fossil Record 2 [54] as our date estimate. More recent publications of fossil Charadriiformes were not included because they either represent specimens that are younger or have not been assigned to families that are represented amongst the extant Charadriiformes (such as Turnipacidae [72]). We assumed that fossil dates represent the earliest occurrence for each group which inevitably resulted in underestimates of clade age. The fossil record of Charadriiformes is amongst the best of the modern bird groups [17] in terms of the numbers of taxa, but many specimens are fragmentary and reliable estimates of divergence dates are dependent on a limited number of exceptional specimens [73]. The phylogenetic affinities of the fossil shorebirds in relation to their extant relatives have not yet been fully established, hence have implicitly assumed that fossil representatives of extant groups would be resolved amongst their living relatives.

Source trees may include estimates of relative branch lengths (e.g., genetic distances). This allows further dating of the supertree but is problematic because different relative estimates are not comparable and cannot be applied directly to the supertree [39]. However, where a source trees shares a node that has an absolute date in the supertree (a node dated from fossil evidence), the relative branch lengths can easily be converted to estimates of age. All taxa in our supertree are either extant, or very recently extinct; hence, the tips of the calibrated supertree should be equidistant from the root of the tree. In source trees where the relative branch lengths are not equidistant from the root, we followed the protocol of Purvis [[39]; p.407–8]. We estimated relative dates using the local molecular clock logic [74] as implemented by Purvis [39] and Bininda-Emonds *et al. *[15]. For example, consider three taxa *A, B, *and *C *where *A *and *B *are sister taxa and *C *is sister to *A *and *B. *The root is dated to 10 million years (myr) from fossil evidence, and independent molecular data provides estimates of divergence based on the number of substitutions per site. The molecular estimates of branch lengths are as follows: *A*, 6 substitutions; *B*, 8 substitutions; *C*, 20 substitutions; *A *and *B *are 11 substitutions from the root. *A *and *B *are therefore separated from their common node by a mean of 7 substitutions. The total length from *A *and *B *to the root is thus 18 substitutions compared to 20 for *C *(a mean of 19). This can be converted to date estimates such that 19 substitutions are equivalent to 10 myr. The dates of the tree are then: ((*A*: 3.68, *B*: 3.68), *C*: 10)). There were no cases where multiple source trees with molecular divergence dates were able to provide estimates for the same node. We estimated relative dates from multiple nodes rather than a single dated node to minimise correlative errors in estimates.

To provide date estimates for all nodes in the tree we employed a pure birth model to date nodes for which absolute and relative dates could not be attained [39]. Pure birth models infer that a clade's age is proportional to the logarithm of the number of species within the clade:

date of daughter = date of ancestor *(log daughter clade size/log parent clade size)

For example, the age of a daughter node that subtends 12 taxa, estimated from its immediate ancestor dated to 20 myr and which subtends 19 taxa is:

20*(log(12)/log(19)) = 16.879

We applied this approach to estimate the ages of daughter nodes based on dates (absolute or calibrated) of ancestral nodes. We had no ancestral node on which to base estimates of the most basal clade. In this case, we rearranged the pure birth formula and calculated the age of the ancestral node from its two daughter nodes, taking the mean as our "best estimate". Finally, to estimate the ages of nodes between daughter and ancestor nodes of known age we spaced the nodes evenly along the branches length [75].

## Authors' contributions

GHT assisted in the design of the study, carried out the phylogenetic analyses and node dating, and drafted the manuscript in partial fulfillment of a doctoral degree at the University of Bath. MAW assisted in the design of the study and with editing and revision of the manuscript. TS assisted in the design of the study, collection of source trees, and editing and revision of the manuscript. All authors read and approved the final manuscript.

## Supplementary Material

Additional File 1 **Estimates of node ages and node support (branch lengths.xls) **Node numbers correspond to figures 2-9. Five types of estimate were used: a) absolute dates from the fossil record; b) absolute dates from molecular point estimates; c) relative dates based on branch length estimates from molecular studies; d) estimates based on a pure birth model (see text for details); and e) even spacing of nodes along branches with daughters and ancestors of known age. The numbers of characters supporting each node are provided (column D), this is equivalent to the number of source trees that share the equivalent node (see text for details). Click here for file

Additional File 2 **Shorebird supertree (50% majority-rule consensus; majrulesupertree.tiff) **Shorebird supertree based on 50% majority-rule consensus of 1496 shortest trees with calibrated branch lengths. Scale bar indicates time from the present in millions of years. Click here for file

Additional File 3 **Shorebird supertree (strict consensus; strictsupertree.tif) **Shorebird supertree based on 50% majority-rule consensus of 1496 shortest trees. Click here for file

Additional File 5 **Source trees (source trees.xls) **A summary of each tree used is given including the data type and main taxa studied. This is a brief summary and the original papers should be consulted for full details. Click here for file

Additional File 6 **MRP matrix (shorebirdMRP.txt) **The MRP matrix used in the shorebird supertree analysis. Click here for file

Additional File 4 **Calibrated supertree (shorebirdsupertree.txt) **The supertree in nexus format including branch length estimates. Click here for file
